# Challenges and Perspectives Regarding the Determination of Gingival Crevicular Fluid Biomarkers During Orthodontic Treatment: A Narrative Review

**DOI:** 10.3390/medicina60122004

**Published:** 2024-12-04

**Authors:** Anamaria Bud, Luminița Lazăr, Maria-Alexandra Mârțu, Timea Dakó, Mircea Suciu, Andreea Vlasiu, Ana-Petra Lazăr

**Affiliations:** 1 Department of Pedodontics, George Emil Palade University of Medicine, Pharmacy, Science, and Technology of Târgu Mures, 38 Ghe. Marinescu Street, 540139 Târgu Mures, Romania; anamaria.bud@umfst.ro (A.B.); vlasiuandreea@gmail.com (A.V.); 2 Department of Periodontology, George Emil Palade University of Medicine, Pharmacy, Science, and Technology of Târgu Mures, 38 Ghe. Marinescu Street, 540139 Târgu Mures, Romania; 3 Department of Periodontology, Grigore T. Popa University of Medicine and Pharmacy Iasi, Universitatii Street 16, 700115 Iasi, Romania; 4 Department of Odontology and Oral Pathology, George Emil Palade University of Medicine, Pharmacy, Science, and Technology of Târgu Mures, 38 Ghe. Marinescu Street, 540139 Târgu Mures, Romania; timea.dako@umfst.ro; 5 Department of Oral Rehabilitation and Occlusology, George Emil Palade University of Medicine, Pharmacy, Science, and Technology of Târgu Mures, 38 Ghe. Marinescu Street, 540139 Târgu Mures, Romania; mircea.suciu@umfst.ro (M.S.); ana.lazar@umfst.ro (A.-P.L.)

**Keywords:** orthodontic treatment, gingival crevicular fluid, biomarkers, review

## Abstract

*Background:* Changes in the positions of teeth occur during orthodontic treatment due to the application of forces that cause restructuring of the periodontal tissue. In the last decade, substantial research has been conducted to detect different biomarkers in the gingival crevicular fluid (GCF) to obtain a better assessment of the periodontal status. *Aim:* The purpose of this review is to describe how the levels of certain biomarkers from the gingival fluid change during tissue remodeling throughout orthodontic treatment. *Materials and methods:* To carry out the purpose of this research, electronic databases were searched using specific keywords, leading to 387 articles, out of which 19 were used in writing this narrative review. A sampling period of the last 10 years was used in selecting the articles. *Results:* The results highlight that the origin of the gingival crevicular fluid is at the gingival blood vessels’ plexus. GCF has a complex composition with differences depending on the periodontal status and the tissue restructuring which takes place in the periodontium. The levels of inflammatory mediators, enzymes, and metabolic products of tissue remodeling in GCF change during orthodontic treatment. Being aware of their specific role, they can provide valuable information about bone remodeling during orthodontic tooth movement. *Conclusions:* By determining the biomarkers in GCF, as an investigative method, clinicians could easily monitor the orthodontic tooth movement, and, subsequently, the treatment period could be shortened and the adverse effects associated with it could be avoided.

## 1. Introduction

Changes in the positions of the teeth arise during orthodontic treatment due to the application of forces, which lead to restructuring of the periodontal tissue. A healthy or stable periodontal status and excellent timing in applying these forces are the keys to a successful orthodontic treatment [1]. The assessment of periodontal status is a valuable step both for patients susceptible to periodontal disease and for patients benefiting from orthodontic or implant treatment [2].

Three of the most used clinical parameters for the evaluation of periodontal status are the periodontal pocket depth (PPD), the clinical attachment loss (CAL), and bleeding on probing (BOP). Besides these, radiographic measurements are also necessary [3]. One of the limitations of these diagnostic methods is the inability to provide pertinent information about the disease progression in the absence of previous records. Highly susceptible individuals who may be at greater risk of bone loss in the future are not being identified by these techniques [4]. To overcome these limitations, considerable research has been conducted in the last few decades to detect different biomarkers in the gingival crevicular fluid (GCF) [5]. The term “biomarker” describes a substance that is objectively measured and evaluated as an indicator of normal biological processes, pathological processes, or pharmacological responses to a drug in an intervention. Molecules, hormones, enzymes, proteins, deoxyribonucleic acid (DNA), ribonucleic acid (RNA), bacterial products, and volatiles are among these biomarkers [6].

Throughout the bone remodeling process, osteoblasts and osteoclasts release multitudinous protein or protein-derived biomarkers, known as bone turnover markers [7]. These markers are predominantly products of bone proteins, especially type I collagen, which go through substantial post-translational modifications during new bone synthesis. Products of bone cells, which reflect the number of certain cells surrounding the bone at a given time, represent other biomarkers involved in the process [4,5,6]. Markers of bone turnover are divided into two groups: markers of bone formation (osteocalcin (OC) and bone alkaline phosphatase (BALP)) and markers of bone resorption (C- and N-Terminal Telopeptides of Type I Collagen (CTx and NTx)) [6,7]. Even if various studies have used bone turnover markers, they are still not widely implemented in clinical orthodontic practice [8,9]. Using them in orthodontics would create new possibilities for comprehending bone remodeling phenomena during treatment. Choosing the correct mechanical loading to shorten the orthodontic treatment period and ward off adverse effects associated with it could imply knowing the mechanisms that take place in the periodontal tissues [10,11,12,13].

The present study aims to discover the relationship between changes in the levels of gingival crevicular fluid biomarkers and the modifications in the periodontal tissue during orthodontic treatment based on recent studies from the literature. This review also aims to discuss how the levels of these biomarkers can be beneficial in the evaluation of the results of orthodontic treatment. This comes from the null hypothesis of the study, which was that there are no changes regarding the levels of gingival crevicular fluid biomarkers during orthodontic treatment.

## 2. Materials and Methods

A review of current literature was undertaken to describe how the levels of certain biomarkers from the gingival fluid change during tissue remodeling throughout orthodontic treatment. Electronic searches were performed in PubMed, Embase, and Scopus to identify and include articles regarding this subject using the keywords “orthodontic treatment”, “gingival crevicular fluid”, “biomarkers”, and “review”. Longitudinal, case–control, retrospective, and prospective studies, as well as reviews, from August 2015 up to August 2024 were included in this research. At first, 387 articles were found. The generated publications were then filtered to remove duplicate articles from all databases. After removing duplicates and articles that did not meet the inclusion criteria of our research, 19 final articles were included in the writing of this review.

## 3. Tissue Restructuring During Orthodontic Treatment

Remodeling in the dental and periodontal tissues occurs due to the application of orthodontic forces, which consequently leads to tooth movement [8]. Due to the orthodontic forces, the alveolar bone suffers two phenomena: on one side, the alveolar bone and periodontal ligament are compressed and subjected to pressure, while on the opposite side, the periodontal ligament is stretched and, thus, put under tension [9].

The vascularity of the periodontal tissue is also affected by the orthodontic forces. On the pressure side, the blood circulation decreases, while on the tension side, it diminishes or remains at the same level. Rapid changes in the blood flow have been observed when the loading force is constant. In this case, biologically active agents such as prostaglandins and cytokines are released. These chemical mediators impact cellular activity differently in the pressure versus tension zones within the periodontal ligament, producing bone resorption on the pressure side and bone formation on the tension side [9,10].

On the compressive side, the force magnitude is linked to varying cellular responses. A strong force leads to the blood flow being cut off and, consequently, to cell death by compression (hyalinization). In the compressed periodontal ligament, there will be no immediate osteoclast differentiation and resorption will be delayed. As a result, it will take 7–14 days for tooth movement to occur. Mild force will only decrease blood flow so that rapid recruitment of osteoclasts from the bloodstream or the periodontal ligament is granted. Due to the resorption of the lamina dura produced by the osteoclasts, tooth movement commences two days after the orthodontic forces have been applied [11].

The onset of acute inflammation is marked by binding and recruiting leukocytes, monocytes, and macrophages into the periodontal ligament through the activated endothelium [10]. The released molecules generate cellular responses around the teeth, providing a favorable environment for tissue deposition or resorption. Cellular signaling pathways are thus initiated, which stimulate changes in the periodontal ligament, resorption, and bone apposition [11]. The transition from an acute process to a chronic and proliferative one involving endothelial cells, fibroblasts, osteoclasts, and osteoblasts happens after a couple of days [12]. The physiological process of bone remodeling or bone turnover is determined by the balance between bone formation, mediated by osteoblasts, and bone resorption, mediated by osteoclasts [13]. These mechanisms must be well controlled because any shortcoming can lead to tissue destruction, manifested by root resorption and the installation of pathological changes characteristic of periodontal diseases [11,13].

Osteoclasts come from macrophages or monocytes, originating in the hematopoietic stem cells of the bone marrow. The complex interaction between macrophage colony-stimulating factor (MCSF), tumor necrosis factor *α* (TNF-*α*), interleukins (IL), parathyroid hormone, vitamin D, and calcitonin mediates osteoclast function [14,15,16]. Bone morphogenetic proteins, whose expression increases at the site where tension is exerted during orthodontic movements, stimulate the differentiation of mesenchymal stem cells into osteoblasts [13]. Prostaglandins and inflammatory cytokines are released concurrently with changes in blood flow and oxygen tension brought on by a small amount of pressure that is maintained for a few minutes. By increasing cyclic adenosine monophosphate (cAMP), inducing many chemical modulators, and promoting cell differentiation within the periodontal ligament, metabolic alterations take place after at least four hours [17]. Activated mature osteoblasts express osteogenic genes that encode several proteins and enzymes (osteocalcin, bone sialoprotein, alkaline phosphatase, and type 1 collagen) which are essential for the formation of the extracellular organic matrix and for its subsequent mineralization [18].

## 4. Biomarkers of Bone Turnover in Gingival Crevicular Fluid

The gingival chorion, which lies beneath the epithelium that forms the soft wall of the gingival sulcus, contains the gingival blood vessel plexus, which is at the origin of the gingival crevicular fluid (GCF). In the presence of periodontal pathology, GCF transudate serum becomes an inflammatory exudate. The fact that neutrophils migrate into the gingival sulcus, even in periodontally healthy conditions, makes it unclear whether GCF can be characterized as an inflammatory exudate or, on the contrary, a physiological transudate [19].

The GCF complex composition differs depending on the periodontal status and the tissue restructuring that takes place in the periodontium, represented by *inflammatory mediators, enzymes, and metabolic products* of tissue remodeling [20]. During the orthodontic treatment, the levels of these components of GCF go through different changes. Knowing the specific role of each of them, they provide valuable information about bone remodeling during orthodontic tooth movement.

### 4.1. Inflammatory Mediators

Prostaglandin E2 (PGE2), which is one of the inflammatory mediators, makes a contribution to stimulating bone resorption, and its level is elevated during orthodontic movements both on the pressure side and on the tension side [21,22,23]. Bone resorption is caused by the activity of the osteoclasts, which are stimulated by a pro-inflammatory cytokine called tumor necrosis factor (TNF α) [24]. The levels of TNF-α increase 1 h and 24 h after the application of orthodontic forces, both in patients with pre-adjusted edgewise appliances (PEA) and in those with self-ligating (SL) systems. In the PEA group, the concentration of TNF-α decreases significantly after 168 h, while in the SL group, this value remains elevated [25]. The immediate increase after one hour in the levels of inflammatory mediators responsible for bone resorption (IL-1β and TNF-α), peaking at 24 h, supports the role of inflammation in initial tooth movement [26].

The changes occurring in the alveolar bone during incisor retraction after the extraction of the first premolars often lead to complications in the form of bone thinning or root resorption because of contact with the palatal cortical plate or the one surrounding the incisive canal. PDL cells produce more activated FAK and PGE2 in response to compressive stimulation. Furthermore, in compressively stimulated PDL cells, a reduction in FAK (focal adhesion kinase) expression that disrupts the integrin complexes through FAK siRNA transfection lowers prostaglandin E2 synthesis. There is limited information regarding the changes in the levels of PGE2 levels during and after CAOT (corticotomy-assisted orthodontic therapy) and PAOO (periodontally accelerated osteogenic orthodontics) techniques [9,27].

The levels of IL-1α, IL-1β, IL-2, IL-6, IL-8, and TNF-α are higher in the fixed lingual appliance compared to the labial appliance, indicating that the lingual appliance might exert greater mechanical stress on the periodontium than the fixed labial appliance [28]. Receptor nuclear factor kappa B ligand (RANKL) and macrophage colony-stimulating factor (M-CSF) are essential for osteoclast formation by rapidly differentiating hematopoietic osteoclast precursors into mature osteoclasts [29,30]. Osteoclast genesis and osteoclast activity are limited by a variety of cytokines and by osteoprotegerin (OPG), another receptor produced by osteoblasts and B lymphocytes, which can inhibit RANKL/RANK interactions and alter the effects of RANKL.

The major determinant of bone mass and bone resorption, which is the RANKL/OPG ratio, is different during orthodontic movements. In the compression zone, RANKL expression is induced and OPG expression is reduced, thus increasing the RANKL/OPG ratio and favoring RANKL-mediated osteoclast genesis [31,32,33]. Conversely, in the tension zone, OPG expression increases, which in turn inhibits RANKL [34,35,36]. The Invisalign system releases an initial force of approximately 1 N in the process of maxillary molar distalization. This force produces an increased concentration of modeling mediators and bone remodeling, both at pressure sites (IL-1b, RANKL) and at tension sites (TGF-1b, OPN) [37].

### 4.2. Enzymes

Alkaline phosphatase (ALP) is an enzyme that is particularly important for the integrity of the periodontal tissue, with a great influence on the formation and regeneration of this tissue. The detection of high levels of ALP in GCF during orthodontic treatment is associated with bone formation [38,39]. Decreased bone formation and increased bone resorption in an orthodontically displaced tooth can be observed by measuring ALP levels [40]. During the orthodontic tooth movement, on day 14 of this process, an increase in ALP expression can be noted. It is more expressed in the tension zone than in the pressure zone with higher values in patients with conventional brackets than in those with self-ligating brackets [41]. Twin Block is the most effective appliance, showing higher levels of ALP activity in the first month, third month, and sixth month of sampling compared to the Clear Block and Forsus groups [42]. Bone mineralization is controlled by ALP, which releases an organic phosphate that precipitates the calcium–phosphate complex in the osteoid matrix. It can also inhibit mineralization by hydrolyzing inorganic pyrophosphate, which prevents the formation of hydroxyapatite crystals [42].

Matrix metalloproteinases (MMPs) are chemokines that can contribute to differential bone remodeling in response to orthodontic forces at both pressure and tension sites [43]. The levels of MMP-1, MMP-2, MMP-3, MMP-8, MMP-9, and MMP-13 increase on both the pressure and tension sides during orthodontic tooth movement [43]. Orthodontic force stimulates MMP-3 expression, possibly through the p38 MAPK pathway, with its strongest signal occurring 24 h after applying orthodontic forces [44]. Recent evidence suggests that MMP-9 seems to be a sensitive marker for periodontal inflammation during orthodontic treatment [45]. MMP-9 has high levels throughout orthodontic tooth movement, appearing already 4 h after the application of orthodontic forces [46]. Furthermore, MMP-13 affects the activity of osteoclasts and bone resorption. This outcome points out that MMP level could be used in the early stages of orthodontic treatment as a marker for active tooth movement.

### 4.3. Metabolic Products

In the GCF of patients undergoing orthodontic treatment, a series of metabolic products can be detected, which are indicators of periodontal tissue remodeling. Throughout the loading periods, chondroitin sulfate levels in GCF increased, with no significant differences between the values at 120 and 180 g forces. Consequently, applying stronger forces was not needed, since there were no differences in bone remodeling activity evaluated biochemically [47].

Pentraxin-3 (PTX-3), also known as gene 14 (TSG-14), stimulated by tumor necrosis factor (TNF), is a 45 kDa glycoprotein with 202 amino acids. After the activation of the orthodontic appliance, patients show an increased level of PTX-3 in the GCF. The maximum level is recorded at 24 h, and the return to initial values occurs after two weeks in children and after one week in adults. This suggests the involvement of PTX-3 in initial orthodontic periodontal remodeling and aseptic inflammation induced by orthodontic forces [48].

Osteocalcin (OC) is a small (5.4 kDa), non-collagenous bone matrix protein that binds calcium. Produced by mature osteoblasts, osteocytes, and odontoblasts, this vitamin K- and D-dependent protein can be found in the extracellular mineralized matrix of the bone and in the blood [49]. In serum or plasma, it moves freely in the decarboxylated form, while in bone, it is present in the inactive carboxylate form [50]. Being described as one of the valuable markers of bone metabolism, OC is also considered a marker of bone turnover due to its part in the recruitment of osteoclasts at the site of bone resorption [51]. The highest values of OC levels were observed between day 7 and 14, after the activation of the orthodontic appliance, with no evident link between the concentration of OC and the time of collection [52]. With the application of orthodontic forces and the occurrence of active bone remodeling phenomena, the level of OC in the GCF goes through changes, starting from the 7th day [53]. Around the area where the tooth movement occurs, on days 14 and 21, a significant statistical increase in the OC levels has been noted [53]. Serum OC may be a sensitive biomarker of alveolar bone loss, as it has been associated with loss of alveolar bone density rather than of alveolar bone height [54].

N-telopeptide (NTx), the amino-terminal peptide of mature type I collagen with attached cross-links, is a specific marker of bone resorption. The NTx molecule is specific to the bone, its formation is mediated by osteoclasts, and it is found in both urine and serum as a stable product of bone degradation. The bone resorption process is correlated with high levels of serum NTx. Comparing the levels of NTx in GCF recorded at 1 h, 1, 7, 14, and 21 days, they gradually increased from day 7 to day 21, with a significant difference observed on days 14 and 21 and maximum levels of NTx in GCF on day 21 [53].

As the disease progresses from gingivitis to periodontitis and pathological mechanisms occur in the periodontal structures, the levels of these biomarkers also change in the GCF. The homeostasis of bone turnover is disturbed during the progression of periodontal disease and leads to irreversible bone resorption [55,56,57]. Taking this into consideration, determining the levels of GCF biomarkers during orthodontic treatment can have a preventive effect on the installation of irreversible periodontal lesions.

GCF is part of the general homeostasis of the body, and its changes may be related to systemic diseases, especially immune-competent, cardiovascular, and post-COVID changes. Considering this, we included articles in which studies were conducted on systemic healthy patients.

The essential findings of studies that evaluated the effects of orthodontic treatment of periodontal structures with the help of some markers are shown in Table 1.

## 5. GCF Harvesting Methods

Gingival crevicular fluid can be used to analyze different biochemical markers due to its ease of sampling, non-invasiveness, and repeatability from the same site. There are three main methods of GCF collection, which will be adapted according to the purpose and objectives pursued: the gingival washing method, collection with the help of capillary microtubes, and the use of sterile absorbent paper strips [20,58,59,60,61].

In the gingival lavage method, a fixed-volume isotonic solution is used to irrigate the gingival sulcus, and then both cells and soluble proteins are collected from the diluted crevicular fluid. The utility of this method can be understood when the objective is to harvest cells from the gingival sulcus. Its disadvantage is that some properties of GCF, such as composition or volume, cannot be evaluated correctly because the dilution factor cannot be precisely determined [20,62].

The accuracy determination of the GCF volume can be obtained by using capillary tubes inserted into the gingival sulcus after adequate isolation and drying. This method allows for precise, site-specific collection of undiluted GCF, but tissue damage can occur due to the length of the collection because it may take more than 30 min [59].

The use of sterile absorbent paper strips is the most used method (Table 1) for collecting GCF samples by inserting them in the gingival sulcus and holding them for 30–60 s. To obtain valid results before collection, the medium must be clean and well isolated, and the paper must be inserted into the gingival sulcus up to the base or until a slight resistance is felt. Samples contaminated with blood or saliva will not be used. The advantages of this technique are speed, ease of use, the possibility of accurate harvesting from a site, and the lack of tissue damage when used correctly [5,59].

Although the volume of collected GCF can be measured by various means, the most common and accurate one is the electronic method. An electronic transducer is used to measure the amount of GCF collected on absorbent paper. This relies on the fact that electronic current flow is affected by the wetness of the paper strip and, thus, provides a digital readout. The biomarkers in the GCF will then be determined by the enzyme-linked immunosorbent assay (ELISA) technique, which is the most widely used method for analyzing the composition of the GCF.

### Limitations of the Study

The results of this study can be seen with certain limitations, especially because of the rather small number of studies analyzed. The present study contributes to increasing reference data in the literature, supporting future research in this field.

## 6. Conclusions

Considering the findings of this study, we concluded that biomarkers in the GCF undergo changes induced by orthodontic forces, which cause bone remodeling. Taking this into consideration, we can say that biomarkers could be used to evaluate the results of orthodontic treatment. Other factors can influence these changes, such as gingival inflammation, occlusal forces, and poor oral hygiene. If these factors are controlled, biomarker levels could be considered as indicators of biological effects produced by orthodontic treatment.

Determining PGE2 and TNF-α levels is beneficial in order to oversee the action of PEA and in SL systems. IL-1α, IL-1β, IL-2, IL-6, IL-8, and TNF-α levels are elevated in patients using a fixed lingual appliance compared to ones using a labial appliance, suggesting that the lingual appliance might exert a greater mechanical stress on the periodontium than the fixed labial appliance. Levels of IL-1b and RANKL are helpful in monitoring the action of Invisalign appliances. MMP levels modify their concentrations in the gingival fluid and point out the early stages of active tooth movement. Determining osteocalcin levels from the crevicular fluid is helpful to notice losses in bone density during orthodontic treatment, while the level of N-telopeptide can offer valuable information about systemic osteoclast activity, specifically while using mandibular protrusive orthodontic appliances.

Orthodontic tooth movement can be monitored by clinicians by determining biomarkers in GCF as an investigative method in order to shorten the treatment period and avoid the adverse effects associated with it. While collecting the GCF can look like a simple method, the determination of biomarkers requires a laborious and expensive paraclinical investigation. With this in mind, we support the idea that developing tests that could be used in the office would aid clinicians and benefit patients.

## Figures and Tables

**Table 1:** Summary of the studies that evaluated levels of biomarkers in the GCF and their main results.

Author/ Year	Studied Marker	GCF Harvesting Method	Main Findings	In Vitro/In Vivo Human/Animal
Shetty et al., 2013, [21]	Prostaglandin E2 (PGE2)	Microcapillary pipette	PGE2 levels peak at 24 h and decline to near-baseline levels 168 h after orthodontic forces are applied.	in vitro, human
Celebi et al., 2013, [23]	Prostaglandin E2 (PGE2) Interleukin-1β (IL-1β)	Paper strips	Ovarian activity may affect orthodontic tooth movement and IL-1β and PGE2 levels in GCF.	In vitro, *animal*
Pramustika et al., 2018, [25]	Tumor necrosis factor-α (TNF-α)	Paper points	TNF-α concentration increases at 1 h and 24 h after orthodontic force application.	In vitro, *human*
Soares Bonato et al., 2022, [26]	Interleukin (IL)–1β, Tumor necrosis factor (TNF)–α	Paper points	IL-1β and TNF-α levels increase immediately after the application of orthodontic forces, reaching a maximum at 24 h.	In vitro, *human*
Gujar et al., 2020, [28]	Interleukin IL-1α, IL-1β, IL-2, IL-6, IL-8, Tumor necrosis factor-α (TNF-α)	Microcapillary pipette	Levels of IL-1α, IL-1β, IL-2, IL-6, IL-8, and TNF-α are higher with fixed lingual appliance compared to labial appliance.	In vitro, human
Nugraha et al., 2023, [31]	Receptor activator of nuclear factor-kappa ligand (RANKL), Osteoprotegerin (OPG)	Paper points	RANKL-OPG expression is enhanced on the tension side but not on the compression side.	In vitro, *animal*
Barbieri et al., 2013, [33]	Activator of nuclear factor-kappa (RANK), Osteoprotegerin (OPG), Osteopontin (OPN), Transforming growth factor ß1 (TGF-ß1)	Paper strips	Levels of mediators of bone resorption (RANK and TGF-ß1) increase and levels of mediators of bone formation (OPG) decrease 24 h after the application of orthodontic forces.	In vitro, human
Castroflorio et al., 2017, [37]	Receptor activator of nuclear factor-kappa ligand (RANKL), Osteoprotegerin (OPG), Osteopontin (OPN), Interleukin 1β (IL-1β), Transforming growth factor ß1 (TGF-ß1)	Paper strips	Invisalign aligners cause an increased concentration of modeling mediators and bone remodeling both at pressure sites (IL-1b, RANKL) and at tension sites (TGF-1b, OPN).	In vitro, human
Kumar et al., 2022, [38]	Alkaline phosphatase (ALP), Lactate dehydrogenase (LDH)	Microcapillary pipettes	ALP and LDH levels increase during the application of orthodontic forces, with considerable differences between the first (alignment and leveling) and the second stage of treatment (retraction).	In vitro, human
Jeyraj et al., 2015, [39]	Alkaline phosphatase (ALP)	Paper filter strips	ALP levels at 0, 1, 7, 14, 21, and 28 days, respectively, indicate a decreasing trend both on the distal side of the canine and on the mesial side of the second premolar.	In vitro, human
Farahani et al. 2015, [40]	Acid and Alkaline phosphatase (ACP and ALP)	Paper strips	Higher values of ALP activity are reported for the mesial (tension) side compared to the distal (compression) side in the distalizing movement of the canine.	In vitro, human
Nassar et al., 2021, [41]	Alkaline phosphatase (ALP), Interleukin-8 (IL-8)	Paper filter strips	Alkaline phosphatase (ALP) activity and interleukin-8 (IL-8) levels are significantly higher in patients with conventional brackets than in those with self-ligating brackets.	In vitro, human
Sundrani et al., 2024, [42]	Alkaline phosphatase (ALP)	Capillary tubes	Twin Block was the most effective appliance, showing higher levels of ALP activity in the first month, third month, and sixth month of sampling compared to the Clear Block and Forsus groups.	In vitro, human
Lin et al., 2021, [43]	Interleukin IL-17, Metalloproteinases MMP-1, MMP-2, MMP-3, MMP-8, MMP-9, MMP-13	Paper strips	Levels of IL-17, MMP-1, MMP-2, MMP-3, MMP-8, MMP9, and MMP-13 increase on both the tension and pressure sides at 24 h and 4 weeks and decrease at 12 weeks after applying orthodontic forces	In vitro, human
Grant et al., 2013, [46]	IL-1beta, IL-2, IL-4, IL-5, IL-6, IL-8, IL-10, TNF-α MMP-9, TIMP-1 șand 2, RANKL, and OPG	Paper strips	The levels of IL-1beta, IL-8, and TNF increase in tension sites starting from 4 h to 42 days after the application of canine distalization force. MMP9 levels increase significantly at pressure sites from 4 h to 7 days after the application of force. TIMP-1 levels increase only 7 days after applying force to the tension side of the canine. RANKL increases only 42 days after orthodontic force application.	In vitro, human
Limsiriwong et al., 2018, [47]	Chondroitin sulphate (CS)	Paper filter strips	Chondroitin sulfate levels increase during loading periods, with no significant differences between values, when applying distalization forces of 120 and 180 g.	In vitro, human
Surlin et al., 2012, [48]	Pentraxin-3 (PTX-3)	Paper strips	PTX-3 levels increase, reaching a maximum after 24 h of activation, and return to the initial value after 2 weeks in children and after 1 week in adults.	In vitro, human
Nassrawin, 2018, [52]	Osteocalcin (OC)	Filter paper strips	The highest values of OC levels were observed between day 7 and 14, after the activation of the orthodontic appliance.	In vitro, human
Yildirim et al., 2023, [53]	Osteocalcin (OC), Type I collagen cross-linked C-terminal telopeptide (ICTP)	Paper strips	Increased levels of OC on the bone formation side and increased levels of ICTP on the bone resorption side were observed at 14 days after the activation of the orthodontic appliance.	In vitro, human

## Data Availability

Not applicable.
